# A randomized trial to evaluate e-learning interventions designed to improve learner's performance, satisfaction, and self-efficacy with the AGREE II

**DOI:** 10.1186/1748-5908-5-29

**Published:** 2010-04-19

**Authors:** Melissa C Brouwers, Julie Makarski, Anthony J Levinson

**Affiliations:** 1 McMaster University, Department of Oncology and Department of Clinical Epidemiology, McMaster University, Hamilton, Ontario, Canada; 2 McMaster University, Department of Oncology, Hamilton, Ontario, Canada; 3 McMaster University, Division of e-Learning Innovation, Hamilton, Ontario, Canada

## Abstract

**Background:**

Practice guidelines (PGs) are systematically developed statements intended to assist in patient, practitioner, and policy decisions. The AGREE II is the revised and updated standard tool for guideline development, reporting and evaluation. It is comprised of 23 items and a user's Manual. The AGREE II is ready for use.

**Objectives:**

To develop, execute, and evaluate the impact of two internet-based educational interventions designed to accelerate the capacity of stakeholders to use the AGREE II: a multimedia didactic tutorial with a virtual coach, and a higher intensity training program including both the didactic tutorial and an interactive practice exercise component.

**Methods:**

Participants (clinicians, developers, and policy makers) will be randomly assigned to one of three conditions. *Condition one, didactic tutorial *-- participants will go through the on-line AGREE II tutorial supported by a virtual coach and review of the AGREE II prior to appraising the test PG. *Condition two, tutorial + practice *-- following the multimedia didactic tutorial with a virtual coach, participants will review the on-line AGREE II independently and use it to appraise a practice PG. Upon entering their AGREE II score for the practice PG, participants will be given immediate feedback on how their score compares to expert norms. If their score falls outside a predefined range, the participant will receive a series of hints to guide the appraisal process. Participants will receive an overall summary of their performance appraising the PG compared to expert norms. *Condition three, control arm *-- participants will receive a PDF copy of the AGREE II for review and to appraise the test PG on-line. All participants will then rate one of ten test PGs with the AGREE II. The outcomes of interest are learners' performance, satisfaction, self-efficacy, mental effort, and time-on-task; comparisons will be made across each of the test groups.

**Discussion:**

Our research will test innovative educational interventions of various intensities and instructional design to promote the adoption of AGREE II and to identify those strategies that are most effective for training. The results will facilitate international capacity to apply the AGREE II accurately and with confidence and to enhance the overall guideline enterprise.

## Introduction

Evidence-based practice guidelines (PGs) are systematically developed statements aimed at assisting clinicians and patients to make decisions about appropriate health care for specific clinical circumstances [1] and to inform decisions made by health care policy makers and clinical managers [2,3]. In systematic reviews, guidelines have been shown to have a modest impact on behavior [4]. However, the potential benefits of their application are only as good as the guidelines themselves [5-7]. To enable differentiation between PGs of varying quality and to advance the PG enterprise, the AGREE (Appraisal of Guideline Research and Evaluation) collaboration was established to facilitate the development of a generic instrument to assess the process of PG development. Using rigorous methodologies of measurement construction [8], the AGREE collaboration produced the original AGREE Instrument released in 2003 [[9]; http://www.agreetrust.org].

As with any new development tool, it was recognized that on-going methodological refinement of the AGREE instrument was required. This led to the establishment of a second international group of researchers, the AGREE Next Steps Consortium. The consortium undertook a program of research with the objectives of strengthening the measurement properties of the instrument, refining some of the items, systematically exploring its utility across stakeholders, and improving the supporting documentation to help users implement the instrument with more confidence. The results of these efforts are the AGREE II [[10-12]; http://www.agreetrust.org]. The AGREE II consists of 23 items grouped into the six original domains: scope and purpose, stakeholder involvement, rigour of development, clarity of presentation, applicability, and editorial independence. Compared to the original AGREE instrument, approximately one-half of the items have been modified, domain structures have been altered, and an extensive restructuring of the supporting documentation, the user's manual, was undertaken. A new seven-point response scale has also been introduced, replacing the original four-point scale. The AGREE II was released at the Guidelines International Network Fall 2009 Colloquium and is ready for use.

Diffusion of the original instrument attests to its wide coverage and acceptance but also highlights the complexity of successfully facilitating the uptake of the revised version. In conducting an analysis of the ISI Web of Science (unpublished), we found 139 citations of the original AGREE paper between its publication in 2003 and December 2008, with numbers increasing every year. Lead authors represented 23 different countries and publications appeared in 95 different peer-reviewed journals -- both specialist and generalist publications. The citations represented a wide spectrum of diseases and disciplines, including cancer, cardiology, diabetes, dentistry, psychiatry, and occupational medicine.

We anticipate the demand for the AGREE II will be as high. We are promoting the AGREE II to a broad constituency and the dissemination plan is international in focus. The target audience includes a variety of stakeholder groups (clinicians, researchers, policy makers, PG developers, system leaders) and, within groups, a range of experience with PGs and the AGREE enterprise (*i.e.*, from novice to expert). Thus, the internet is a key medium for our knowledge translation and exchange (KTE) strategy. However, dissemination alone, even with a primed and interested audience, is not sufficient to maximize the application and use of the AGREE II.

Thus, we wish to explore educational interventions and leverage technical platforms to accelerate the process. E-learning (internet-based training) provides a potentially effective, standardized, and cost-efficient model for training in the use of AGREE II. A recent meta-analysis and systematic review of 201 studies by Cook *et al. *showed large effect sizes for internet-based instruction (clinical and methodological content areas) with health-profession learners [13]. Most of the studies considered knowledge outcomes and found evidence of a substantial benefit. Those studies reporting a skills outcome, however, also found a very large effect size for e-learning interventions. The findings held true in subgroup analyses comparing different learner types, contexts, topics and outcomes. Thus, e-learning appears to be a promising, effective, practical, and efficient KTE technique to consider in our context, and we will test two interventions aimed at facilitating the application of the AGREE II.

Key evidence-based principles exist that underpin the development of technical training and multimedia learning to which we will adhere. The Instructional Systems Development framework, including the ADDIE (analysis, design, development, implementation, and evaluation) model of instructional development will serve as our approach in the design and refinement stages of our intervention [14]. The work by Clark *et al. *will inform the structure and specific content types that will be incorporated [15-18]. Narration choices, contiguous labeling, and the use of graphics will follow the principles of multimedia learning [17,19]. Principles derived from cognitive load theory will also be taken into consideration in the design of the educational interventions [16,20].

In a meta-analysis and systematic review of instructional design variations in web-based learning, Cook *et al. *found that increased interactivity, practice exercises, repetition, and feedback were associated with improved learning outcomes [13]. However, while the evidence base underpinning the efficacy and design principles of internet-based training materials are well established, there remain questions regarding the optimal application of these principles for particular interventions. For example, both worked examples (demonstrations) and practice exercises with feedback have been shown to be effective training methods [17]. Yet some evidence suggests that novice learners may benefit more from worked examples, and expert learners more from practice [16,18]. Moreover, many recommended instructional design interventions such as interactivity, practice exercises, or repetition may take longer to develop, and also take up more of the learners' time, potentially leading to less efficient training. In developing an optimal on-line training intervention for the AGREE II, we also aim to study some of these key instructional design variables and time-on-task.

Our research objectives are: to design and refine an on-line AGREE II training program comprised of a multimedia didactic overview tutorial; to design and refine an on-line, interactive AGREE II training program, comprised of the overview tutorial plus an interactive practice exercise with feedback module; to compare the two interventions against a standard control (access to static PDF version of the user's manual) and to evaluate learners' performance (distance function to experts, pass/fail rate), satisfaction, self-efficacy, mental effort, and time-on-task with the AGREE II; and to compare how previous experience with PGs and the AGREE II influence these effects.

Two core research questions are considered: Compared to the passive learning of the materials, does an on-line training program, with or without a practice exercise, improve learners' performance and increase learners' satisfaction and self-efficacy/-confidence with the AGREE II and AGREE II user's manual? Are there differences across the outcome measures between the two educational intervention groups? Are these differences influenced by learners' experiences with PGs or the AGREE II?

## Methods

This study is funded by the Canadian Institutes of Health Research and has received ethics approval from the Hamilton Health Sciences/Faculty of Health Sciences Research Board Ethics approval (REB #09-398; Hamilton, Ontario, Canada).

### Study design

A single factorial design with three levels of educational intervention is proposed. The levels are:

### Didactic tutorial

Participants assigned to this training program condition will receive access to a password-protected website. They will receive a brief (five-minute) multimedia didactic tutorial with an overview of the AGREE II conducted by a 'virtual coach' or avatar. The tutorial is under program control with forced linear progression in sequence with the screens advancing automatically, although the participant may pause the tutorial at any time. Following the tutorial, the participant is granted access to the AGREE II user's manual and is instructed to review the manual before proceeding to the test PG.

### Tutorial with practice exercise

Participants assigned to this training condition will receive access to a password-protected website. They will be provided with the same didactic tutorial as the previous condition before being granted access to the user's manual as above. They will then be presented with a practice PG to appraise using the AGREE II training tool and will be asked to answer each AGREE II item in turn. Upon entering their AGREE II score, participants will be given immediate feedback on how their score compares to the mean of four experts. If their score falls outside a predefined range, participants will receive formative feedback to guide the appraisal process. At the conclusion of their review, participants will receive an overall summary of their performance in appraising the practice PG compared to expert norms before proceeding to the test PG.

### Passive learning

Participants assigned to the passive learning will receive static PDF copies of the AGREE II for review before proceeding to the test PG. Passive learning participants will serve as our control group.

### Sample Size

The primary analysis involves one-way analysis of variance (ANOVA) comparisons of the AGREE II performance score profiles of the three study group participants with the performance score profiles of AGREE II experts. This will be measured by the sum of squared deviations (SS) distance function. To avoid untenable assumptions regarding the relative size of the intermediate group mean, we simplify calculations by focusing on the power for testing differences in mean SS between the passive learning condition and either of the intervention groups, which represents a strong *a priori *comparison of the least and most effective interventions. Previous research has found the effect size of e-learning in comparison to no intervention to be large ranging from 1.13 to 1.50 [16-18]. Our intent is to estimate a more conservative effect size. Thus, with 20 participants per group, a one-sided test will have at least 80% power to detect an advantage of as little as ± 0.79 standard deviations for either of the intervention groups compared to the passive learning group. To account for potential missing data, we will include up to 25 participants per group for a total of 75 participants in the study.

### Materials and instruments

#### Guidelines

Eleven PGs have been selected from the National Guidelines Clearinghouse http://www.guidelines.gov, CMA Infobase http://www.cma.ca/index.cfm/ci_id/54316/la_id/1.htm, and Guidelines International Network http://www.g-i-n.net/ directories for this study. One PG will serve as the practice PG for those assigned to the *tutorial + practice exercise *condition, and ten will serve as the test PGs in the study. Criteria for the PG search included: English-language PGs, PGs produced from 2002 onward, PGs with core text of 50 pages or less, and PGs targeting one of three clinical areas: cancer (n = 4), cardiovascular disease (n = 4), and critical care (n = 2). From the eligible candidates, and to choose a sample of ten test PGs, we selected PGs that reflected a range of quality on the Rigour of Development domain of AGREE II. Although we are not interested in the differences in PG topic as a primary factor, we want variability in clinical topic to make our findings more generalizeable.

#### AGREE II

The AGREE II consists of survey items and a user's manual.

#### Items

The AGREE II consists of 23 items grouped into six domains: scope and purpose, stakeholder involvement, rigour of development, clarity of presentation, applicability, and editorial independence. Items are answered using a seven-point response scale (strongly disagree-strongly agree). Standardized domain scores for PGs are calculated by summing scores across the appraisers and standardizing them as a percentage of the possible maximum score a PG can achieve per domain. This method enables the construction of a performance score profile permitting direct comparisons across the domains or items. The AGREE II concludes with two global measures answered using a seven-point scale: one targeting overall quality and the second targeting intention to use the PG.

#### User's manual

The AGREE II also comprises supporting documentation, referred to as the AGREE II user's manual. The user's manual provides details for each of the 23 items, including: explicit descriptors for the different levels on the seven-point rating scale; a description that defines each concept underlying the item and specific examples; direction on common places to look for the information and common terms or labels that represent to the concept(s); and guidance on how to rate the item, including specific criteria and considerations.

#### Learners' scale

In addition to the primary outcome of accuracy on the PG rating scale using AGREE II, secondary measures will also be collected: learner satisfaction, self-efficacy, mental effort, time-on-task, learner satisfaction, and self-efficacy with the training intervention will be measured using a seven-point scale. Mental effort will be measured on a seven-point scale, using self-report, and correlated with performance outcome to determine the cognitive efficiency metric [16]. Self-reported time-on-task related to the training time will be collected and checked against server logs. A time efficiency metric will also be determined, correlating time-on-task with performance outcome.

#### AGREE II experience scale

The Experience Scale, used originally with the AGREE Next Steps Project, will be modified and applied here. This scale asks participants about their experience in the PG enterprise (as developers, evaluators of PGs) and their experiences using the AGREE II tool (to facilitate development, reporting, and evaluation of PGs).

#### Expert norms

Expert norms will be compared to participants' AGREE II performance score profiles. Expert norms will be derived by members of the AGREE Next Steps research team who will appraise the PGs used in this study (n = 10). Mean standardized scores will be used to construct the expert performance score profiles.

#### Participants and procedures

Seventy-five participants will be recruited to participate in this study. Participants will reflect the range of potential PG and AGREE II users: clinicians, developers, and researchers, administrators, and policy makers. Because we found no differences in patterns of evaluation among user stakeholder group in the development work leading up to the release of the AGREE II [[10], http://www.agreetrust.org], we have not included stakeholders as a variable of interest.

Participants will be recruited from various sources, including: methodologists, clinicians, administrators, and policy makers involved in formal PG development programs; first authors of published PGs in the National Guideline Clearinghouse, CMA Infobase, and Guidelines International Network directories; professional directories and professional associations reflecting different stakeholder groups; clinical and health service researcher trainees; and the Guideline International Network community. A strong list of international collaborators will assist in our recruitment efforts. Candidate participants will be e-mailed a letter of invitation to participate in this study. After screening for their eligibility, participants will be randomly assigned by the research coordinator using a computer-generated randomization sequence to one of the three educational intervention groups. They will receive access to an individualized password-protected web-based study platform. There, participants will participate in the intervention to which they were assigned, complete an evaluation of one of the ten test PGs using the AGREE II, and complete a series the post-test Learner's Scales. Participants will be blinded to the other conditions.

### Analyses

#### Performance -- distance function

Our primary outcome for performance will be a measure of distance in AGREE II item and domain rating profiles of the participants versus rating profiles of experts. The distance function will be calculated as the sum of the squared deviations (SS) between expert scores and participant's scores, summed over AGREE II items (SSi) and, alternatively, domains (SSd). Such a measure offers a precise and integrated summary of similarity over the whole profile of responses, and it provides a standard quadratic weighting of errors, consistent with other widely used measures of agreement, such as weighted kappa. Since the SS is typically skewed, we will use its square root in analysis. A series of one-way ANOVA tests will then be conducted to examine differences in distance function as a function of educational intervention.

#### Performance -- pass/fail

A pass/fail algorithm has been designed and pilot tested to categorize AGREE II users as meeting minimum performance competencies with the tool. This algorithm has been pilot tested and refined and is currently used by the Capacity Enhancement Program of the Canadian Partnership Against Cancer (CPAC) to hire appraisers to participate in the evaluation of more than 800 cancer PGs using the AGREE II. The pass/fail algorithm will be used to compare competency rates across the educational intervention using X^2 ^statistics.

#### Learner's scales

A series of one-way ANOVA tests will be conducted to examine differences in participants' satisfaction, self-efficacy, cognitive effort, and time-on-task scores as a function of educational intervention.

#### Test guideline ratings -- AGREE II scores

For exploratory purposes, a series of one-way ANOVA tests will be conducted to examine differences in participants' standardized AGREE II domain scores on the test PGs as a function of educational intervention.

#### Guideline and AGREE II experience

For exploratory purposes, measures of PG and AGREE II Experience captured at time one will be used a covariate in the analyses proposed above.

## Discussion

This project represents one of two initiatives of the AGREE A3 Consortium. We hope to complete this initiative in 2010. Our study findings will better inform KTE initiatives related to PG standards and evaluation, as well as the literature on instructional design and optimal training program design to balance learning and performance outcomes with time efficiency. In particular, our study will help determine the effectiveness and efficiency of practice exercises related to guideline review training, as well as learner satisfaction with web-based learning in this context.

## Competing interests

The authors declare that they have no competing interests.

## Authors' contributions

MCB conceived of the concept and design of the originally funded proposal, drafted and revised this manuscript, and has given final approval for the manuscript to be published.

JM contributed to the design of the originally funded proposal, contributed substantially to the revisions of the manuscript, and has given final approval for the manuscript to be published. AJL contributed to the design of the originally funded proposal, contributed substantially to the revisions of the manuscript, and has given final approval for the manuscript to be published.
